# Regulation of PD-L1 Protein Expression by the E3 Ubiquitin Ligase GP78

**DOI:** 10.3390/cimb47100829

**Published:** 2025-10-09

**Authors:** Madhumita Chatterjee, Julio M. Pimentel, Jun-Ying Zhou, Thamarahansi Mugunamalwaththa, Zhe Yang, Avraham Raz, Gen Sheng Wu

**Affiliations:** 1 Departments of Oncology and Pathology, Karmanos Cancer Institute, Wayne State University School of Medicine, Detroit, MI 48201, USA; chatterjeem845@macomb.edu (M.C.); zhouj@karmanos.org (J.-Y.Z.);; 2 Cancer Biology Program, Wayne State University School of Medicine, Detroit, MI 48201, USA; jupimentel@health.ucsd.edu; 3 Department of Biochemistry, Microbiology and Immunology, Wayne State University School of Medicine, Detroit, MI 48201, USA; hansi@wayne.edu (T.M.);

**Keywords:** GP78/AMFR, PD-L1, ubiquitination, degradation

## Abstract

Immune checkpoint inhibitors (ICIs), including PD-L1 inhibitors, have been approved by the FDA for the treatment of cancers; however, only a small number of cancer patients benefit from these ICIs. Furthermore, the development of drug resistance to this type of treatment is often inevitable. The mechanisms of resistance to PD-L1 inhibitors can be attributed, in part, to an incomplete understanding of the regulation of PD-L1 protein expression. In this study, we identified the role of the E3 ligase GP78, also known as the Autocrine Motility Factor Receptor (AMFR), in the regulation of PD-L1 protein levels. We show that GP78 physically interacts with PD-L1, which is confirmed by IP and Western blotting and is supported by molecular modelling using AlphaFold2. Our modeling studies predict that the interface amino acids of the Ig1 domain of PD-L1 interact with the RING domain and a β-hairpin preceding the CUE domain of GP78. The crystal structure of the PD-1/PD-L1 complex reveals that the interaction with PD-1 is mediated by the Ig1 domain of PD-L1. Furthermore, proteasomal degradation of PD-L1 has been observed via GP78-mediated K48-linked ubiquitination, indicating a key regulatory role for GP78 in the downregulation of PD-L1. Because GP78 expression is inversely correlated with PD-L1 levels in cancer, these findings may have clinical implications for predicting tumor immune evasion and patient response to PD-1/PD-L1 blockade therapies. Taken together, these findings identify a previously unknown mechanism by which GP78 targets PD-L1 for ubiquitination and subsequent degradation in cancer cells, and suggest that blocking the interaction between PD-L1 and PD-1 by an E3 ligase is a novel strategy to improve immunotherapies for cancer patients.

## 1. Introduction

The immune checkpoint ligand Programmed cell death ligand 1 (PD-L1), also known as CD274 and B7-H1, is a type I transmembrane glycoprotein that belongs to the immunoglobulin superfamily [1]. PD-L1 is expressed on the surface of antigen-presenting cells, tumor cells, or non-transformed cells. Elevated PD-L1 protein levels have been detected in many different cancers, including breast, colorectal, and non-small cell lung cancer (NSCLC) [2], and are associated with a poor prognosis. Furthermore, PD-L1 expression is regulated at the transcriptional level by several transcription factors that are involved in oncogenic and inflammatory signaling pathways [2]. The activation or mutation of several transcription factors, including Myc, HIF1/2α, NF-κB, MAPK, PTEN/PI3K and EGFR, which are components of multiple oncogenic signaling pathways, leads to the upregulation of PD-L1 mRNA [3]. Studies have shown that elevated levels of PD-L1 mRNA, mediated by the IFN-γ-induced JAK/STAT1/IRF1 pathway, are associated with immune evasion in melanoma cells [1]. Post-translational modifications of PD-L1, including glycosylation, phosphorylation, and ubiquitination, are key regulators of protein stability and protein–protein interactions [3]. PD-L1 suppresses the immune response by interacting with Programmed cell death 1 (PD-1) (CD279) in T cells, thus inhibiting their activation and allowing cancer cells to evade immune surveillance [1,2]. PD-L1 is heavily glycosylated, showing only N-linked glycosylation in the extracellular N-terminal domain of PD-L1, which contains the N-X-T/S motif and shows a high level of glycosylation in N35, N192, N200, and N219 [4]. The oncogenic behavior of these N-linked glycosylated residues in PD-L1 is associated with tumorigenesis, as studies using 4NQ mutants have shown increased anti-tumor immunity [4]. Consistently, PD-L1 glycosylation is required for the interaction between PD-L1 and PD-1 in breast cancer cells [4]. Therefore, promoting PD-L1 degradation to prevent the PD-L1-PD-1 interaction is a feasible strategy to develop more effective cancer immunotherapies.

Glycoprotein 78 (GP78), also known as the autocrine motility factor receptor (AMFR), is an E3 ubiquitin ligase. GP78 is a resident transmembrane protein of the endoplasmic reticulum (ER) involved in the ER-associated degradation (ERAD) of misfolded proteins, which is necessary for maintaining cellular homeostasis [5,6]. Overexpression of autocrine motility factor (AMF) and the AMF receptor (AMFR) is often associated with cancer and poor clinical outcomes. In breast cancer, elevated GP78 expression and the formation of AMF-AMFR complexes are negatively associated with clinical outcomes and are implicated in breast cancer progression [7]. Upregulation of GP78 expression has also been reported in bladder carcinoma [8], while histochemical analyses in colorectal cancer patients have revealed an association between higher GP78 expression and lower survival rates and cancer recurrence [5]. Similarly, high AMFR expression in stage I non-small cell lung cancer is associated with poor clinical outcomes [9]. Beyond these clinical correlations, GP78 also plays a critical role in protein quality control and signaling. It binds to ERAD substrates such as the cystic fibrosis transmembrane conductance regulator (CFTR) and apolipoprotein B (APOB) and targets them for ubiquitination, followed by proteasomal degradation [10,11]. GP78 self-ubiquitination is mediated by the 25 kDa protein containing the tripartite motif (TRIM25) for proteasomal degradation [12]. A recent study from our lab has reported that ERK activation is mediated by GP78 through the degradation of DUSP1, resulting in EGFR-dependent proliferation and invasion of cancer cells [13].

In this study, we describe a previously unreported mechanism of PD-L1 degradation, preceded by GP78-mediated ubiquitination. This post-translational modification depends on a physical interaction between GP78 and PD-L1, as demonstrated by immunoprecipitation (IP) assays and molecular modeling of the GP78-PD-L1 complex. Our study reveals that the core interface residues of the GP78-PD-L1 complex overlap with those involved in the binding of PD-L1 to PD-1. These findings suggest that the downregulation of the PD-L1 protein by GP78-mediated degradation can lead to blockade of the PD-L1-PD-1 interaction, allowing the immune system to target and attack cancer cells more effectively.

## 2. Materials and Methods

Cell lines and cell culture. HEK293T, OV433, and MDA-MB-231 cells were purchased from the American Type Culture Collection (ATCC) (Manassas, VA, USA). These cells were grown in Dulbecco’s modified Eagle’s medium (DMEM) containing 1% penicillin/streptomycin and 10% fetal bovine serum (FBS) and maintained at 37 °C in a humidified atmosphere of 5% CO_2_.

The reagents used are as follows: Bio-Rad (Hercules, CA, USA): 30% acrylamide/Bis (1610156). Calbiochem (San Diego, CA, USA): Protein G PLUS/Protein A-Agarose (IP05). Cell Signaling (Danvers, MA, USA): PD-L1 Rabbit mAb (13684), anti-AMFR antibody (9590S), DYKDDDDK (Flag) mAb (14793S), K48-linkage polyubiquitin antibody (4289), anti-mouse IgG, HRP-linked secondary antibody (7076S), anti-rabbit IgG, HRP-linked secondary antibody (7074S), and normal rabbit IgG (2729). EMD Millipore (Burlington, MA, USA): Ub-K63 monoclonal antibody (05-1308). GE Healthcare (Waukesha, WI, USA): Penicillin-streptomycin (SV30010). Gibco (Mount Laurel, NJ, USA): Trypsin-EDTA and DMEM (11995-065). Invitrogen (Carlsbad, CA, USA): Blasticidine (R210-01), Lipofectamine 2000 (11668-027), Lipofectamine RNAiMAX (13778-075), V5 monoclonal antibody (R960-25), goat anti-mouse IgG Alexa fluor 680 secondary antibody (A21058), and goat anti-rabbit IgG Alexa fluor 680 secondary antibody (A-21109). Santa Cruz (Dallas, TX, USA): GP78 (sc-293371), anti-HA antibody (F7) (sc-7392), and normal mouse IgG (c-2025). Sigma-Aldrich (St. Louis, MO, USA): FBS (F0926), Z-Leu-Leu-Leu-al (MG132) (C2211), puromycin (P9620), protease inhibitors (5892970001), phosphatase inhibitors (4906845001), β-actin antibody (A1978), and ANTI-FLAG^®^ M2 affinity gel (A2220-1ML). ThermoFisher (Waltham, MA, USA): West Pico PLUS chemiluminescent substrate (34580), and Sample buffer (50-196-784). Dharmacon (Lafayette, CO, USA): siGenome Human AMFR siRNA-SMART pool (M-006522-01-0005) contains the following sequences: (1) GCAAGGAUCGAUUUGAAUA, (2) GGAGCUGGCUGUCAACAAU, (3) GAGGACUGCUCAUGUGAUU, and (4) CGAGCUGGCUGCCGAGUUU. siGenome non-targeting siRNA pool # 1 (D-001206-13-05) contains the following sequences: (1) UAGCGACUAAACACAUCAA, (2) UAAGGCUAUGAAGAGAUAC, (3) AUGUAUUGGCCUGUAUUAG, and (4) AUGAACGUGAAUUGCUCAA.

### 2.1. Plasmids and Cloning

Full-length human GP78 cDNA was inserted into pCDNA6-V5, as described [12]. The pCMV-PD-L1 flag plasmid was purchased from Sino Biological Inc (Wayne, PA, USA). (HG10084-CF). PRK-HA-Ub, tagged with hemagglutinin-ubiquitin (HA-Ub), was purchased from Addgene (17608) (Watertown, MA, USA). OV433-L-gpva2-shRNA GP78 and OV433-PL01-con shRNA cells were prepared using shRNA constructs against hGP78 that were purchased from Open Biosystems (Lafayette, CO, USA): va1 V2LHS_11875 NM_001144 shRNAmir Hs Lentiviral pGIPZ RHS4430-98521426, va2 V2LHS_14183 NM_001144 shRNAmir Hs Lentiviral pGIPZ RHS4430-98715204, va3 V2LHS_208794 NM_001144 shRNAmir Hs Lentiviral pGIPZ RHS4430-99291935, and va4 V2LHS_206751 NM_001144 shRNAmir Hs Lentiviral pGIPZ RHS4430-99294626. To generate OV433 cells with GP78 knockdown, control and V2LHS_14183 constructs were transfected into OV433 cells and selected with puromycin. A single colony was then collected, analyzed, and confirmed by Western blotting.

### 2.2. Transfection

For plasmid DNA transfection, mammalian cells were 80% confluent at the time of transfection and transfected with Lipofectamine 2000 following the manufacturer’s instructions. Cells were treated with 10 μM MG132 for 4 h prior to 24 h before being harvested. For siRNA transfection, mammalian cells were grown to 50% confluency and transfected with Lipofectamine RNAiMAX following the manufacturer’s instructions. Cells were harvested after 48 h.

### 2.3. Generation of MDA-MB-231-pcDNA6-V5-hGP78 and MDA-MB-231-pcDNA6-V5 Vector Stable Cell Lines

MDA-MB-231 cells were transfected with pcDNA6-V5-hGP78 or pcDNA6-V5-Vector. After 24 h, cells were washed with PBS and selected with Blasticidin at 10 μg/mL. The process was repeated every 3 days until separate colonies were visible and had grown in the presence of blasticidin. The colonies were picked and the GP78 overexpression clones were verified by Western blotting.

### 2.4. Immunoprecipitation (IP) Assay

IP was performed as described elsewhere [13]. Specifically, cells were lysed in 1X NP-40 lysis buffer supplemented with protease inhibitors on ice. After centrifugation at 4 °C for 12 min, cell extract supernatants were collected, and 500 μg of protein lysate was used for the IP assay. For the GP78 pull-down, 0.8 μg of mouse V5 antibody or control mouse antibody was used to bind to 20 μL of protein A/G agarose beads and 500 μg of protein lysate was added. The next day, the beads were centrifuged at 5000× *g* and washed three times with 1X NP-40 wash buffer, followed by the addition of 40 μL of 2X Laemmli buffer. Then they were boiled and Western blotting was performed. For PD-L1 pull-down, M2 Flag affinity agarose gel beads (40 μL) were used, and immunoprecipitation was performed according to the manufacturer’s protocol.

### 2.5. Molecular Model for the GP78-PD-L1 Interaction

The structure of the PD-L1-GP78 complex was predicted using the AlphaFold server powered by AlphaFold 2 (https://deepmind.google/technologies/alphafold/ accessed on 16 September 2025) [14]. The full-length amino acid sequence of human PD-L1 (residues 1-290, UniProt ID: Q9NZQ7) and the sequence of human GP78 (residues 309-643, UniProt ID: Q9UKV5) were used as input in paired complex mode. Multiple structural models were generated using the default random seed settings. The top-ranked models were selected using a combination of quantitative and structural criteria: a mean per-residue pLDDT (predicted Local Distance Difference Test) ≥ 70 for structured regions and a mean PAE (predicted Aligned Error) < 5 Å across predicted interfaces to ensure confident domain placement. In addition to these numerical thresholds, we visually inspected domain compartmentalization to confirm that the predicted ER-lumen and cytosolic domains were orientated plausibly and were consistent with expected biology. Structural visualization and superposition analysis of the PD-L1-GP78 complex with the PD-1-PD-L1 crystal structure (PDB code: 3BIK) were performed using PyMOL v2.5.5 software.

### 2.6. Analysis of GP78 and PD-L1 Protein Expression in the Human Protein Atlas

Publicly available immunohistochemistry (IHC) data for GP78 (CAB026381) and PD-L1 (CAB080537) were obtained from the Human Protein Atlas (https://www.proteinatlas.org accessed on 16 September 2025). Data plots show the percentage of patient samples (a maximum of 12 per cancer type) with high, medium, or low protein expression, grouped by tissue of origin. Representative HPA breast cancer IHC images were selected to illustrate GP78 and PD-L1 expression patterns.

#### Western Blotting

The preparation of whole-cell lysates and Western blot analysis have been previously described [15]. Cells were lysed in 1X NP-40 lysis buffer and centrifuged at 4 °C for 12 min. A total of 50 μg of protein lysate was used for gel electrophoresis on a 10% denaturing polyacrylamide gel. After transferring and blocking with a 2% Casein solution, the membrane was washed and then incubated with the appropriate primary antibody. It was subsequently labeled with fluorescently labeled or HRP-conjugated secondary antibodies. Antibody binding was detected using the Li-COR Odyssey Imaging system or by HRP protein detection systems. Densitometric analyses of protein bands were performed using ImageJ1.54p (NIH), with signal intensities normalized to the corresponding actin loading control for each blot.

In vivo ubiquitination. HEK293T cells were transfected with PRK-HA-Ub, pCMV-PD-L1-Flag, and either pcDNA6-V5-hGP78 or pcDNA6-V5-Vector. After 24 h, cells were lysed with 1X RIPA lysis buffer. For in vivo ubiquitination of PD-L1, we followed the procedure described previously. Briefly, the cell lysates were boiled at 100 °C for 10 min, cooled, and then sonicated. After centrifugation, the supernatants were saved, and protein concentrations were measured. For the immunoprecipitation assay, 500 μg of protein lysates were used and diluted to 1:1000 with dilution buffer [15]. The lysates were then incubated with M2 Flag affinity gel agarose beads at 4 °C in a rotor overnight, following the manufacturer’s instructions. The antigen–antibody complex bound to the agarose beads was washed, and Western blotting was performed.

## 3. Results

### 3.1. GP78 Levels Are Negatively Correlated with PD-L1 Protein Levels

**Figure 1:**
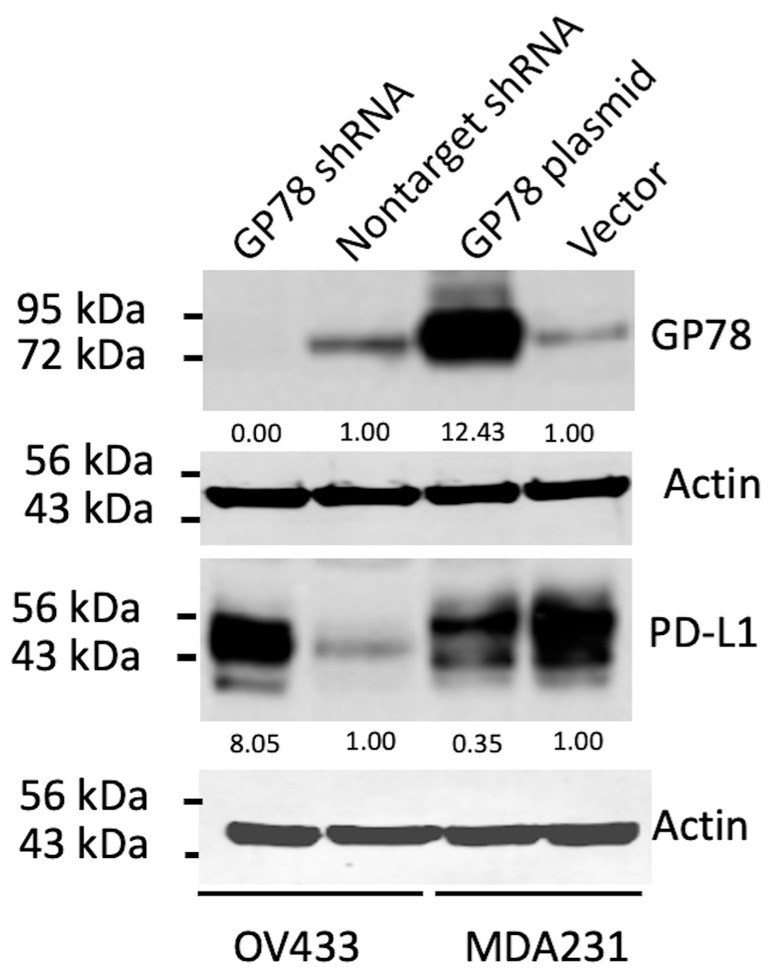
GP78 negatively regulates PD-L1 protein expression. MDA231 cells were stably transfected with the GP78 expression plasmid or a control vector, while OV433 cells from ovarian cancer were used to stably knock down GP78 by transfecting GP78 shRNA or nontarget shRNA. GP78 and PD-L1 levels were assessed by Western blotting using total cell lysates. Densitometric analyses of protein bands normalized to the corresponding actin loading control for each blot (below).

During the course of studying the regulation of phosphatase DUSP1 by GP78 [13], we generated MDA231 cells overexpressing GP78 by stably transfecting them with a vector expressing GP78 or a control vector. We also generated GP78 knockdown OV433 cells by stably transfecting them with short hairpin RNA (shRNA) against GP78 or with a non-targeting control shRNA. We chose these two cell lines because MDA231 cells express low levels of GP78. whereas OV433 cells express relatively high levels of GP78. Figure 1 shows that stable overexpression of GP78 in MDA231 cells decreases PD-L1 protein levels compared to cells transfected with the control vector, with densitometric analysis revealing a ~0.65-fold reduction relative to the control. In contrast, stable knockdown of GP78 in OV433 cells results in increased levels of PD-L1 protein with approximately 8 times compared to cells transfected with a non-targeting shRNA. Thus, these results suggest that GP78 negatively regulates PD-L1 protein expression in cancer cells.

### 3.2. GP78 Promotes PD-L1 Ubiquitination

**Figure 2:**
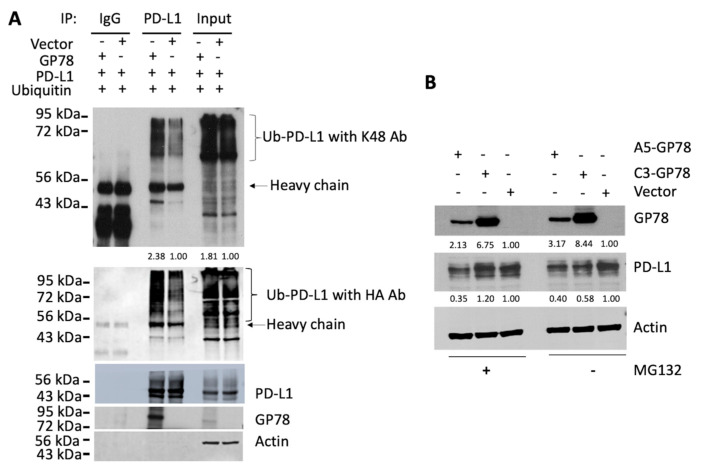
A role for GP78 in the ubiquitination and degradation of PD-L1. (**A**) 293T cells transfected with the indicated plasmids were subjected to immunoprecipitation (IP) with IgG or FLAG antibody, followed by immunoblotting (IB) with K48 antibodies (upper panel), HA antibodies (middle panel), and PD-L1, GP78, and Actin antibodies (lower panel). The lower right panel shows the input PD-L1 protein. (**B**) MDA231 cells with vector control (vector) and two overexpression clones of GP78, C3-GP78 and A5-GP78, were treated with and without MG132 (10 μM) for 4 h. The resulting cells were harvested to assess the expression of GP78 and PD-L1 by Western blot analysis. Actin was used as a loading control. Densitometric analyses of protein bands normalized to the corresponding actin loading control for each blot (below).

As GP78 is an E3 ubiquitin ligase, its negative regulation of the PD-L1 protein suggests that GP78 may promote PD-L1 ubiquitination and subsequent degradation, thus decreasing the level of the PD-L1 protein. To test this possibility, we cotransfected 293T cells with plasmids expressing ubiquitin-HA and PD-L1-FLAG in the absence or presence of GP78-V5. The resulting cells were harvested and subjected to immunoprecipitation (IP) using a FLAG antibody, followed by Western blot analysis with antibodies against ubiquitin, K48, HA, PD-L1, GP78, and actin. Figure 2A shows an increase in ubiquitinated PD-L1, detected by K48 antibodies and HA antibodies, along with a decrease in total PD-L1 in cells transfected with GP78 compared to those transfected with the empty vector. The increased ubiquitination of PD-L1 in cells transfected with GP78 suggests that GP78 functions as an E3 ubiquitin ligase for PD-L1 and that GP78-mediated ubiquitination of PD-L1 may play a crucial role in regulating its protein levels in cancer cells. No ubiquitination of PD-L1 was observed with K63 antibodies. Therefore, our data suggest that GP78-mediated ubiquitination of PD-L1 is a K48-dependent link that mediates PD-L1 degradation. To further define proteasomal degradation of PD-L1 after its polyubiquitination by GP78, MDA231 vector control and two clones overexpressing GP78, C3-GP78 (high GP78 overexpression) and A5-GP78 (low GP78 overexpression), were treated with or without MG132, and GP78 and PD-L1 levels were examined by Western blotting. Figure 2B shows that, without MG132, PD-L1 levels were higher in vector control cells than in MDA-MB-231 C3 GP78 and MDA-MB-231 A5 GP78 (Figure 2B). By contrast, MG132 treatment increased PD-L1 levels in MDA231-C3-GP78 compared to the same cells in the absence of MG132 treatment, which were comparable to vector control cells treated with MG132, while MDA231 did not have a significant impact on PD-L1 in MDA231-A5-GP78 cells, probably due to low overexpression of GP78.

### 3.3. GP78 Physically Interacts with PD-L1

**Figure 3:**
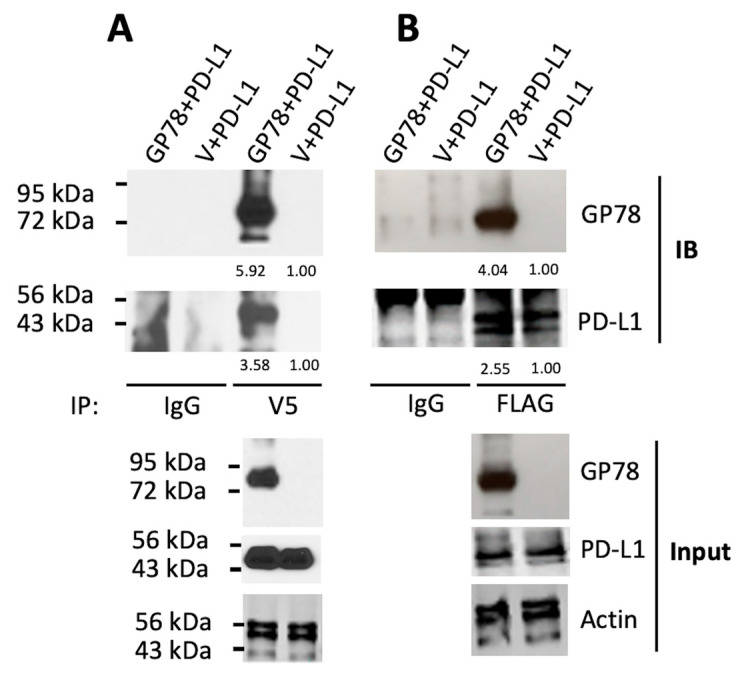
GP78 interacts with PD-L1. 293T cells were cotransfected with PD-L1-FLAG and GP78-V5 or a control vector. Cell lysates were prepared for immunoprecipitation (IP) with V5 antibody (**A**), FLAG antibody (**B**), or normal mouse IgG. A Western blot with an anti-V5 antibody was performed to detect GP78, and an anti-FLAG antibody was used to detect PD-L1 (upper panel). The input was shown (lower panel). Densitometric analyses of protein bands normalized to the corresponding actin loading control for each blot (below).

The physical interaction between an E3 ubiquitin ligase and its substrate is a prerequisite for ubiquitinated protein degradation. To test whether GP78 interacts with PD-L1, we generated pcDNA6-GP78-V5 and pCMV-PD-L1-FLAG constructs and transfected them into 293T cells. HEK293T cells were used in our study (Figure 2) due to their high transfection efficiency, which enables consistent protein expression and provides a controlled, reproducible system for investigating protein–protein interactions. Figure 3A shows that GP78 was co-immunoprecipitated with PD-L1 when whole cell lysates were incubated with a V5 antibody. Reciprocally, PD-L1 was co-immunoprecipitated with GP78 when cell lysates were incubated with a FLAG antibody (Figure 3B). Similar results were obtained in MDA231 cells. Thus, the co-IP of PD-L1 and GP78 strongly suggests a physical interaction between the two proteins, which has not been previously recognized.

### 3.4. The PD-L1/GP78 Interaction Is Supported by Structure Modeling

**Figure 4:**
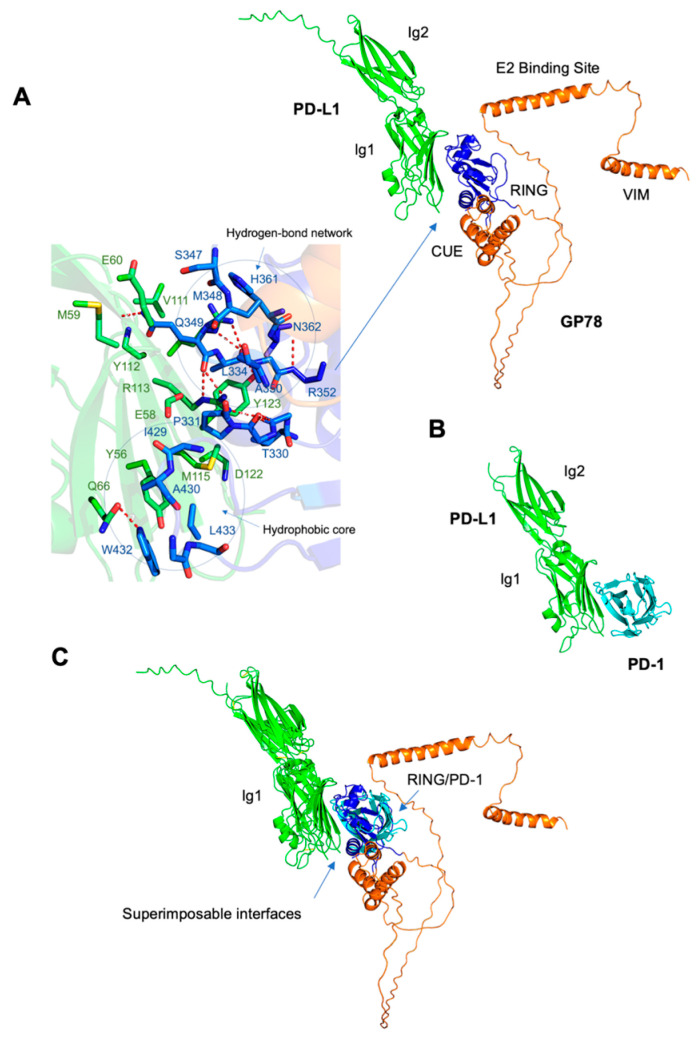
The PD-L1/GP78 interaction was supported by structure modeling. Predicted structure of the PD-L1/GP78 complex. (**A**) AlphaFold was used to predict the structure of the PD-L1/GP78 complex. PD-L1 is colored green and GP78 is colored orange. GP78 consists of four major domains: RING (residues 341–379), CUE (residues 456–498), E2 binding site (residues 574–612), and VIM (residues 622–640). PD-L1 contains two immunoglobulin-like domains: Ig1 (residues 19–132) and Ig2 (residues 133–231). The Ig1 domain of PD-L1 was predicted to interact with the RING domain and a β-hairpin preceding the CUE domain of GP78 (blue). An inset shows the detailed interactions at the interface. Residues involved in the interaction are represented as sticks, with those in PD-L1 shown in green and those in GP78 shown in blue. Hydrogen bonds are depicted as red dotted lines. The interaction between PD-L1 and GP78 is mediated by a network of hydrogen bonds and hydrophobic contacts. (**B**) Crystal structure of the PD-1/PD-L1 complex (PDB code: 3BIK). The structure reveals that the interaction with the PD-1 receptor (green) is mediated by the Ig1 domain of PD-L1 (cyan). (**C**) Structural superposition of the PD-1/PD-L1 and PD-L1/GP78 complexes.

To provide supporting evidence for the physical interaction between PD-L1 and GP78, we used AlphaFold to predict the formation of the PD-L1/GP78 complex. Figure 4A shows the structure of the PD-L1/GP78 complex predicted by AlphaFold, with PD-L1 in green and GP78 in orange. GP78 consists of four major domains: RING (residues 341–379), CUE (residues 456–498), the E2 binding site (residues 574–612), and VIM (residues 622–640). In contrast, PD-L1 contains two immunoglobulin-like domains: Ig1 (residues 19–132) and Ig2 (residues 133–231). The Ig1 domain of PD-L1 was predicted to interact with the RING domain and a β-hairpin preceding the CUE domain of GP78 (blue). The crystal structure of the PD-1/PD-L1 complex (PDB code: 3BIK) revealed that the interaction with PD-1 (green) is mediated by the Ig1 domain of PD-L1 (cyan) (Figure 4B), which is crucial to maintaining peripheral immune tolerance and plays a significant role in immune homeostasis. Furthermore, the structural superposition of the PD-L1/PD-1 and PD-L1/GP78 complexes was evaluated. We found that the binding modes of the two complexes are superimposable, with the Ig1 domain of PD-L1 using the same surface to interact with GP78 and PD-1 (Figure 4C). In our study, AlphaFold prediction was used as supporting evidence for the physical interaction between GP78 and PD-L1 proteins, which was demonstrated using a co-IP assay. The Co-IP assay is widely applied in cancer research to study protein–protein interactions, while AlphaFold offers a structural model of their binding and probable domains (interface amino acids) that might be involved in the formation of the protein complex.

### 3.5. Expression Levels of the GP78 and PD-L1 Protein in Human Cancers

**Figure 5:**
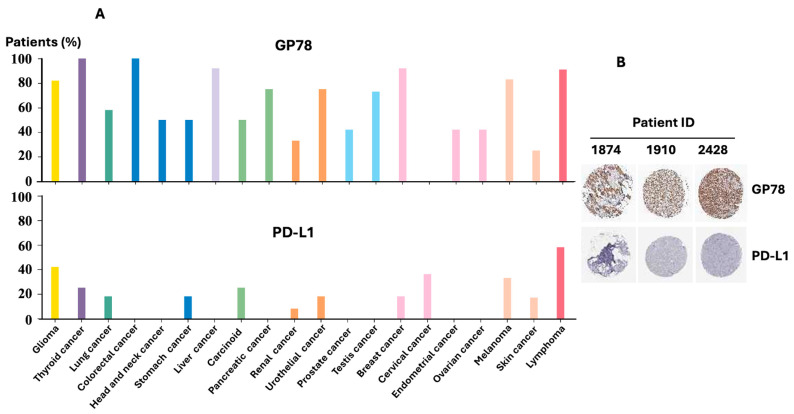
GP78 and PD-L1 protein expression in human breast cancer samples from the Human Protein Atlas. Protein expression data were obtained from the Human Protein Atlas (https://www.proteinatlas.org accessed on 16 September 2025) using the antibodies CAB026381 (GP78) and CAB080537 (PD-L1). (**A**) Top: Summary of the expression of the GP78 protein in cancer types. Bottom: Summary of the expression of the PD-L1 protein in all types of cancer. For each cancer, color-coded bars indicate the percentage of patient samples (a maximum of 12 per cancer type) with high and medium protein expression levels, while a white bar represents low or undetectable expression levels. Cancer types are grouped according to their tissue of origin. (**B**) Representative immunohistochemistry (IHC) images of breast cancer tissues stained for GP78 (CAB026381) and PD-L1 (CAB080537) obtained from the Human Protein Atlas.

To explore the clinical relevance of the expression patterns of GP78 and PD-L1 in various types of cancer, we examined publicly available immunohistochemistry (IHC) data from the Human Protein Atlas (HPA) using antibodies CAB026381 (GP78) and CAB080537 (PD-L1). Cancer types are grouped according to their corresponding normal tissues of origin. Figure 5A (bottom) shows that high or medium expression of PD-L1 was most prominent in glioma, lung, stomach, urothelial, and cervical cancers, while colorectal, pancreatic, prostate, ovarian, and liver cancers predominantly showed low or undetectable PD-L1 staining. In particular, in cervical cancer, the expression of GP78 was not detected, but the expression of PD-L1 was high. In contrast, in both colorectal and liver cancers, the expression of GP78 was high, but PD-L1 was undetectable. In general, GP78 protein expression is inversely correlated with PD-L1 expression. To further demonstrate this inverse relationship at the tissue level, Figure 5B presents representative IHC images from breast cancer specimens. Breast tumors with high GP78 staining showed low or absent PD-L1. Together, these data highlight distinct expression patterns of GP78 and PD-L1 in various cancers, supporting an inverse association between the levels of these two proteins in human tumors.

## 4. Discussion

PD-L1/PD-1 immune checkpoint blockade is at the forefront of research aimed at developing clinically feasible treatments for cancer patients. Our study showed that GP78 physically binds to and targets PD-L1 for degradation. A direct interaction between GP78 and PD-L1 was observed in HEK293T cells (Figure 3A,B), and this interaction was supported by molecular modeling experiments using AlphaFold (Figure 4A–C). Among the four domains of GP78-RING (residues: 341–379), CUE (residues: 456–498), E2 binding site (residues: 574–612), and VIM (residues: 622–640), the RING functions to confer E3 ubiquitin ligase activity, which facilitates the transfer of ubiquitin to the target substrates from ubiquitin-conjugating enzymes (E2s) thereby tagging the target proteins for proteasomal degradation [16]. The region corresponding to β-hairpin loop between the RING finger and the GP78 CUE domain is responsible for its E3 ligase activity [16]. PD-L1 contains two extracellular domains, the IgV domain (also called Ig1, residues 19–132) and the IgC domain (also called Ig2, residues 133–231) [17]. Our study predicted an interaction between the Ig1 domain of PD-L1 and the RING domain, together with a β-hairpin preceding the CUE domain of GP78 (blue, Figure 4A). The interface amino acids demonstrate extensive stabilization through hydrophobic interactions and hydrogen bonds. Hydrophobic interactions involve Ile54, Tyr56, Val68, Met115, and Tyr123 of PD-L1, and Val328, Pro331, Phe424, Ile429, Trp432, and Leu433 of GP78. Six hydrogen bonds are formed at the interface, including those between Glu60 and Gln349, Gln66 and Trp432, Arg113 and Gln349, Tyr123 and Ala351, and Arg125 and Ala350. Studies showed that the Ig1 domain of PD-1 (green) interacts with the Ig1 domain of PD-L1 (cyan, Figure 4A) in the crystal structure of the PD-1/PD-L1 complex (PDB code: 3BIK). This binding is crucial because it sends inhibitory signals to suppress T lymphocyte proliferation and decrease cytokine production, thus weakening the immune system and allowing tumors to bypass immune surveillance [18]. Our study demonstrated that the superposition of the PD-L1/PD-1 and PD-L1/GP78 complexes ensured that the binding modes of the two complexes were superimposable, suggesting that PD-L1 utilizes the same surface of the IgG1 domain to bind to PD-1 or GP78. Several core interface residues in PD-L1 are shared between the complexes, including Ile54, Tyr56, Gln66, Arg113, Met115, Tyr123, and Arg125. These residues, predicted by AlphaFold, provide useful hypotheses for potential interaction sites, and future experimental studies are needed to confirm their functional relevance. More mutation analysis will be required to assess the effects of PD-L1 interface residues on tumor growth.

PD-L1 inhibitors, such as monoclonal antibodies or small molecules, directly block PD-L1 from binding to the PD-1 protein [19]. Li et al. reported a novel drug design strategy using PROTACs (Proteolysis Targeting Chimeras), which recruit E3 ligases to ubiquitinate a substrate of interest and target it for degradation by the proteasome. PROTAC function by forming a ternary complex that involves the PROTAC molecule, the protein of interest (ligand), and an E3 ligase, all linked through an optimized chemical linker [20]. More studies are needed to determine whether GP78 can be recruited more efficiently to target PD-L1 for degradation, thus enhancing immunotherapy strategies for cancer treatment.

PD-L1 is known to be ubiquitinated by various E3 ligases [21], but the underlying mechanism involving the glycosylation status of PD-L1 remains largely unknown. PD-L1 is highly N-glycosylated, a modification necessary for its stabilization and binding to PD-1, which suppresses immune responses in cancer [22]. Recent studies have described the formation of ternary complexes involving mPNGase, AMFR (GP78), and other ER-associated degradation components, which provide a mechanistic framework in which deglycosylation precedes ubiquitination [23,24]. By screening a two-hybrid yeast library using mPNGase as bait, a previous study identified AMFR as one of the interacting proteins that forms a complex with mPNGase [23]. The cytoplasmic tail of AMFR was found to be involved in this interaction [23]. The formation of a ternary complex composed of the E3 enzyme AMFR, p97, mPNGase, and mHR23B was proposed to bridge the ER and the proteasome [24]. According to this model, the first step involves the deglycosylation of the substrate by mPNGase, followed by ubiquitination. In the next step, the misfolded, deglycosylated, and ubiquitinated protein is channelled to the proteasome by mHR23B [24]. Thus, further investigation will be necessary to determine whether the removal of N-glycosylated residues from PD-L1 is directly or indirectly promoted by GP78 in cancer cells, providing a deeper understanding of the events that occur before ubiquitination.

PD-L1 homeostasis is regulated by its proteasomal degradation. Our study not only demonstrated the physical interaction of PD-L1 with GP78 but also showed that PD-L1 polyubiquitination by GP78 occurred through a K48-linked ubiquitin chain (Figure 2). GP78 is an ER-associated protein, while PD-L1 is primarily expressed on the cell surface. It is unclear how these two proteins, located in different compartments, interact. We speculate that PD-L1, after synthesis, is transported to the plasma membrane through the secretory pathway. It is known that cells can utilize various signaling mechanisms to retrotranslocate proteins from their native cell organelles, and one of the ways is by exposing the degradation signal (degron) of the substrate and subsequent recognition by E3 ubiquitin ligase complexes during a misfolding event [25,26]. E3 ligase is typically anchored to the endoplasmic reticulum and mediates substrate recognition via degron, followed by ubiquitination and subsequent degradation. The substrate and E3 ligase, located in different organelles, can utilize molecular glues that bind to both the E3 ligase and the substrate, bringing them into close proximity for a series of degradation events to occur. Studies have shown that adaptor proteins are recruited to bring the cognate substrate into close proximity to Cullin-RING ligases (CRLs) for the formation of the E3 ligase complex [27].

While further study is needed to confirm their interaction and subsequent impact on PD-L1 protein levels, our results demonstrate that GP78 alters the steady-state levels of endogenous PD-L1 compared with control vector–transfected cells, which maintain consistent basal PD-L1 expression. Specifically, GP78 overexpression decreased PD-L1 levels in MDA-MB-231 cells, whereas GP78 knockdown in OV433 cells increased PD-L1 protein levels (Figure 1). These findings provide indirect evidence that GP78 regulates the stability of PD-L1. Furthermore, ubiquitination assays (Figure 2) demonstrate that GP78 promotes K48-linked ubiquitination of PD-L1, a modification well established for targeting proteins for proteasomal degradation. It is established that proteins bearing K48-linked polyubiquitin chains are primarily targeted for degradation by the ubiquitin-proteasome system (UPS) [28], whereas K63-linked polyubiquitin chains are involved in endocytosis [29]. In addition to regulating PD-L1 protein expression, it is possible that GP78 may also impact PD-L1 mRNA, which will be addressed in future studies.

## 5. Conclusions

Taken together, our study has identified PD-L1 as a substrate of GP78, which promotes the polyubiquitination of PD-L1 and its subsequent degradation. It remains to be determined whether GP78 is involved in the deglycosylation of PD-L1. It has been reported that after entering the ER-associated degradation (ERAD) pathway, some glycoproteins undergo deglycosylation before being ubiquitinated by an E3 ligase, resulting in proteasomal degradation. A study revealed that the E3 ligase Murine double minute 2 (MDM2) facilitates the deglycosylation of PD-1 by the glycosidase NGLY1 before the ubiquitination event [30]. However, as discussed earlier, the E3 ligase can participate in the ubiquitination event through the recognition of the degron on the substrate, and it cannot be ruled out that deglycosylation must occur before ubiquitination.

Furthermore, GP78 binding to AMF has been demonstrated to enhance MMP3 expression, resulting in increased cell motility and invasiveness [31]. Therefore, an in vivo approach to the development of immunotherapeutic strategies can involve targeting AMF binding to its monoclonal antibody, as previously reported [32], thus inhibiting GP78-AMF signaling and allowing GP78 to interact more readily with PD-L1 for degradation. We speculate that the degradation of PD-L1 by GP78 E3 ligase reduces the abundance of PD-L1 protein on the surface of tumor cells. As a result, the binding of PD-L1 to PD-1 on T cells is reduced, reducing the inhibition of T cells, thus enhancing tumor immune surveillance and ultimately improving the overall anti-tumor immune response. However, if tumors express a low level of GP78, those tumors can express a high level of PD-L1 due to GP78-mediated downregulation. Tumors with high levels of PD-L1 are believed to be more responsive to PD-1/PD-L1-based immunotherapies. Thus, the negative regulation of PD-L1 protein levels by the E3 ubiquitin ligase GP78 can enhance cancer immune surveillance and also serve as an alternative biomarker to predict whether cancer patients can benefit from PD-1/PD-L1-based cancer immunotherapies. These two possibilities require further investigation.

## Data Availability

The original contributions presented in this study are included in the article. Further inquiries can be directed to the corresponding author.
